# Molecular Typing of *Clostridium botulinum* Isolated from Chili Pepper Preserves During a Botulism Outbreak

**DOI:** 10.3390/foods14244189

**Published:** 2025-12-06

**Authors:** Sara Arnaboldi, Roberto Benevenia, Paola Monastero, Luigi Bornati, Giulia Magagna, Marina Nadia Losio, Guido Finazzi

**Affiliations:** 1 Food Safety Department, Istituto Zooprofilattico Sperimentale della Lombardia e dell’Emilia Romagna (IZSLER), Via A. Bianchi 9, 25125 Brescia, Italy; sara.arnaboldi@izsler.it (S.A.);; 2 Department of Veterinary Science, University of Parma, Strada del Taglio 10, 43126 Parma, Italy; 3 National Reference Centre for Emerging Risks in Food Safety (CRESA), Istituto Zooprofilattico Sperimentale della Lombardia e dell’Emilia Romagna (IZSLER), Via Celoria 12, 20133 Milan, Italy

**Keywords:** foodborne disease, Whole Genome Sequencing, source identification, botulinum neurotoxins, BoNT/B, food safety

## Abstract

Foodborne botulism is a potentially fatal disease caused by *Clostridium botulinum* neurotoxins (BoNTs). These spore-forming bacteria are ubiquitous in the environment and can contaminate various food products, especially raw vegetables. During the preparation of home-made preserves, favorable conditions of anaerobiosis, temperature, salinity, and pH can lead to spore germination and toxin production. BoNTs can reach neuromuscular junctions where they block the release of acetylcholine. In this study, we present a case of foodborne botulism associated with the consumption of chili peppers preserve containing BoNT/B. The isolated strains were characterized through Whole Genome Sequencing, confirming the strains involved in the outbreak. This work increases the understanding of the epidemiology and the ecology of *C. botulinum*, highlighting the importance of raising medical awareness and making timely clinical diagnoses for the effective management of botulism outbreaks.

## 1. Introduction

*Clostridium botulinum* is a Gram-positive spore-forming anaerobic bacterium, ubiquitous in the environment. Its spores can contaminate different raw or cooked foods, causing foodborne botulism (FB), a life-threatening paralytic disease [1,2]. In suitable conditions of temperature (>10 °C), salinity (NaCl concentration < 5–10%), pH (≥4.6), and O_2_ absence, the spores can germinate into vegetative cells and produce botulinum neurotoxins (BoNTs), the most potent biological toxins known [3]. BoNTs are classified from A to H and are mainly produced by *C. botulinum* groups I to IV, but they can also be synthetized from strains of *C. butyricum*, *C. sporogenes*, and *C. baratii* [4,5,6,7]. After ingestion, BoNTs are rapidly absorbed and inhibit acetylcholine release at the neuromuscular junction, resulting in neuromuscular paralysis [8]. Symptoms of foodborne botulism typically appear within 12 to 72 h after the ingestion of the contaminated food. The disease often begins with nausea, vomiting, and abdominal pain and progresses to more severe symptoms such as ptosis, diplopia, dysphagia, dysphonia, and limb paralysis, which may rapidly extend to the respiratory muscles, leading to respiratory failure and death. According to the World Health Organization, botulism is fatal in 5–10% of cases, and early clinical diagnosis is fundamental but extremely challenging due to the non-specific nature of the initial symptoms [9]. Treatment usually requires intensive care and the administration of antitoxin, which can halt the progression of paralysis and improve prognosis [10]. Supportive care may also involve mechanical ventilation, management of autonomic dysfunction, and treatment of secondary infections [11]. Complete recovery usually takes months or years [12].

In Italy, botulism has been notifiable since 1975 and was classified as a Class I disease in 1990, requiring immediate reporting [13,14]. Notifications by the Local Health Authority (LHA) and timely laboratory analysis are essential to confirm the diagnosis and promptly identify the toxin involved, enabling rapid administration of antitoxin. Despite substantial knowledge regarding *C. botulinum* pathogenesis and clinical management, a significant knowledge gap persists concerning the investigation of foodborne botulism outbreaks when no leftover contaminated food is available. In particular, the use of Whole Genome Sequencing (WGS) to retrospectively establish a genetic link between clinical isolates and strains recovered from other jars of the same home-made batch remains underreported. Addressing this gap is crucial for reconstructing outbreak dynamics and guiding timely public health interventions. In this study, we described the management of an outbreak of FB caused by the consumption of a contaminated home-made chili pepper preserve. Three individuals consumed the same product; only one person developed severe symptoms and all survived despite the substantial delay in diagnosing FB.

## 2. Materials and Methods

### 2.1. Case Description and Epidemiological Investigation

On 4 May 2025, during a family lunch, three out of four household members ate chili pepper home-made preserve canned in oil; all three developed gastroenteric symptoms.

- Patient 1 (male): On 5 May, patient reported abdominal cramps and intestinal constipation after the consumption of the preserve oil.
- Patient 2 (male): On 6 May, patient experienced weakness, vomiting, and dysphagia, after the consumption of a large quantity of chili peppers. He consulted the General Practitioner the same day; five days later, he was admitted to the Emergency Room and diagnosed with intestinal blockage. Between 12 and 20 May, he developed progressive neurological symptoms, including diplopia, ptosis, dysphagia, and diplegia. On 20 May, he was admitted to the Geriatric Department of the Niguarda Hospital (Milan, Italy), where foodborne botulism was suspected. Rectal swab and intestinal washing samples were collected on 31 May and sent to Istituto Zooprofilattico Sperimentale della Lombardia e dell’Emilia Romagna (IZSLER, Brescia, Italy) for *C. botulinum* and BoNT detection.
- Patient 3 (female): On 7 May, patient reported vomiting and headache after the consumption of the preserve oil.

Following confirmation of the *C. botulinum* presence in Patient 2, the Local Health Authority (LHA) investigated the outbreak source. The suspected food, a chili pepper preserve canned in oil (containing Habanero peppers, oil, and garlic), was entirely eaten by the three patients and thus was not available for further analysis. Nine additional home-made preserves from the household were collected and sent to IZSLER for analysis (Table 1): two cans containing chili peppers (not Habanero) and oil (samples 1–2), six cans containing Habanero peppers and oil (samples 3–8), and one can containing Habanero peppers, oil, and garlic (sample 9).

The preserves were prepared by cleaning fresh chili/Habanero peppers with a wet cloth, drying for at least three hours at room temperature, cutting, and placing in clean cans with oil; garlic was added to two cans. Cans were closed, boiled in water for 30 min, cooled in water, and stored at room temperature until consumption.

After the results on the food samples, the LHA decided to also collect the stool samples of Patients 1 and 3 to assess the presence of *C. botulinum* and BoNTs.

### 2.2. Laboratory Investigations

#### 2.2.1. *C. botulinum* and BoNT Detection

Both food and biological samples were analyzed following the CNRB30 and CNRB31 protocols provided by the Italian National Reference Centre for Botulism (CNRB) [15,16]. The CNRB30 protocol was used to detect and identify the botulinum toxins using the mouse bioassay in compliance with Directive 2010/63/EU on the protection of animals used for scientific purposes, whereas the CNRB31 protocol was applied to detect the target genes *bont/A*, *bont/B*, *bont/E*, *bont/F* (encoding botulinum toxins), and *4gyrB* (used as process control, CP) using a multiplex real-time PCR.

Briefly, for BoNT detection, samples were homogenized in a phosphate-gelatin buffer solution (0.4% Na_2_HPO_4_ - Sigma-Aldrich, St. Louis, MI, USA, 0.2% gelatin - Sigma-Aldrich, St. Louis, MI, USA, pH 6.2) and injected intraperitoneally in mice, who were observed for 3 days for clinical signs of botulism (shaggy fur, fatigued abdominal breathing, leg weakness, total paralysis, and death caused by respiratory failure). The control mice were injected with specific antitoxins.

For BoNT gene detection, samples were enriched in tryptone peptone glucose yeast extract (TPGY) broth, incubated at 30 °C in anaerobiosis for 24 and 96 h. Then, DNA was extracted from 1 mL of enrichment and real-time PCR was performed. CP detection was used to validate the process.

#### 2.2.2. Genomic Characterization of the Isolated Strains

*C. botulinum* strains were isolated from the positive samples for Whole Genome Sequencing (WGS) analysis. Briefly, the enrichment broths were pasteurized at 70 °C for 10 min and then plated on Egg Yolk Agar (EYA) and incubated in anaerobiosis at 37 °C for 48 h [15]. The culture of each sample was dissolved in 180 µL of G2 buffer (QIAGEN, Hilden, Germany) and 20 µL of lysozyme. After 30 min of incubation at 30 °C, DNA was extracted using the EZ2 Connect automated extractor (QIAGEN, Hilden, Germany) and the DNeasy^®^ Blood & Tissue kit (QIAGEN, Hilden, Germany), in accordance with the protocol for bacterial DNA extraction. Libraries were prepared using the Illumina DNA Prep Kit and Nextera DNA CD Indexes Kit (Illumina, San Diego, CA, USA). The genome was sequenced on the MiSeq Illumina System using the Illumina MiSeq Reagent Kit v2 (500-cycle, Illumina, San Diego, CA, USA), with a theoretical coverage of 75%. The reads quality was assessed using FastQC v0.72; Trimmomatic v0.39 was then used to remove adapters, SPAdes v3.13 to assemble the genomes, and Prokka v1.14.6 to annotate the genomes. The genomes were inspected with Abricate v1.0.1 searching for anti-microbial resistance (AMR) genes, with ResFinder and Megares used as databases. The minimum identity percentage was 90%, while the minimum coverage was 60%. The strains’ sequence type (ST) was assigned using the public databases for multi-locus sequence typing (PubMLST). Finally, the CFSAN Single Nucleotide Polymorphism (SNP) pipeline was applied to check the differences between strains by performing SNP typing [17]. The parameters used in the analysis were as follows: minimum depth at SNP position = 10×; minimum relative depth per SNP position = 10%; minimum distance between SNP = 10 bp; minimum SNP quality = 30; minimum read mapping quality = 25; minimum Z score = 1.96.

## 3. Results

### 3.1. C. botulinum and BoNT Detection

The real-time PCR detected the *bont/B* gene in both the rectal swab and the intestinal washing samples of Patient 2, while the *bont/A, bont/E,* and *bont/F* genes were not detected. Stool samples from Patients 1 and 3 were negative for *C. botulinum* genes; this result is in accordance with the mild symptoms and the fast recovery.

**Figure 1:**
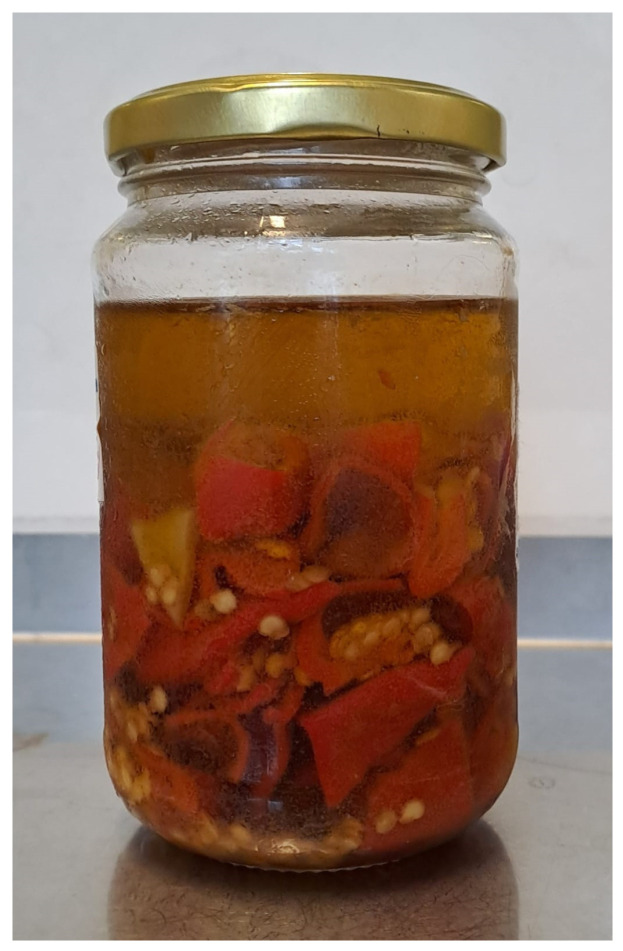
Home-made chili pepper preserve that resulted positive for *C. botulinum* detection.

Analysis of the suspected food samples showed that five out of nine home-made chili pepper preserves (samples 1, 5, 6, 7, and 9, Figure 1) tested positive by real-time PCR. All PCR-positive preserves were hermetically sealed and not consumed by the patients. Patient 2, who developed severe symptoms, consumed the entire Habanero pepper preserve, while Patients 1 and 3 with mild symptoms consumed the oil but not the chili peppers. These results suggest that the outbreak source was the Habanero peppers contained in the entirely consumed preserve, prepared contemporaneously with the positive preserves.

Mouse bioassay performed on the five real-time PCR-positive food samples and was negative in all cases.

### 3.2. Isolate Characterization

Six *C. botulinum* strains were isolated from the human samples (*n* = 2, strains isolated from rectal swab and intestinal washing) and from the food products (*n* = 4, strains isolated from samples 4, 6, 7, and 9) positive by real-time PCR.

WGS of the six isolates was performed with an average coverage of 99.8%. The cluster passing filter of the run was 81%, with a Q30 of 70 and an error rate of 2.36.

The mean reads quality was high (Q-score = 30.5), except from the rectal swab isolate, which was consequently excluded from this study. The number of reads obtained was from a maximum of 531,793 (sample 7) to a minimum of 348,708 (sample 9). The number of contigs ≥ 500 bp was from 21 (sample 7) to 25 (sample 6), and the N50 was from 425,101 (intestinal washing) to 626,666 (sample 4), as shown in Table 2. Overall, the obtained genome lengths ranged between 3,813,179 bp (samples 4 and 6) and 3,860,024 bp (sample 7 and intestinal washing).

The L50 values—the number of contigs covering at least 50% of the assembly—were consistent, ranging from 2 to 4. The results for the longest contigs were also reliable, ranging from 1.25 to 1.60 Mbp, apart from sample 6, which had a value of 0.69 Mbp. Overall, the genome assemblies were robust and well-structured, with total lengths consistent with those of *C. botulinum*, permitting further analysis to identify STs and AMR genes (Table 3).

AMR gene analysis identified only *cfr(C)* in all five strains tested via the Megares database; no additional AMR gene was detected using the ResFinder database.

MLST indicated that the isolated strains belonged to two different STs; sample 4 was assigned to ST29, while samples 6, 7, and 9 and the isolate from the intestinal washing of the Patient 2 belonged to ST53, confirming that the strain contained in the chili pepper preserves was the source of the food poisoning. The SNP typing confirmed that the isolates from samples 6, 7, 9, and from the intestinal washing are highly related (≤129 differences, Table 4), with sample 7 (Habanero peppers, oil) and the intestinal washing having zero differences.

## 4. Discussion

Foodborne botulism is a rare but potentially severe disease occurring in Italy at a rate of 20–40 cases per year [18].

Home-made preserves are the main source of FB, reflecting regional culinary traditions. In this study, the implicated food was a chili pepper preserve in oil, highlighting the ongoing risk associated with improperly processed home-made products. A peculiarity of BoNTs and bacteria that colonize food is that the contamination often goes unnoticed. Unpleasant odors are not always produced when *C. botulinum* grows in the food, and the bubbles caused by gas production are not always visible. Proper food preparation and storage are therefore important to avoid intoxication [19,20]. In Italy, the LHA has issued detailed guidelines to minimize the risk of food poisoning when preparing home-made preserves, given the long Italian culinary tradition of preparing these products [21]. Proper washing and pre-treatment of vegetables are critical steps to reduce contamination risk in the initial preparation: vegetables should be washed under running water or with a water–vinegar solution and blanched before further processing. Moreover, the toxins produced by *C. botulinum* are heat-labile, thus boiling the preserve at 100 °C for 10 min would be sufficient to inactivate toxins and safely consume the preserve after long-term storage [22].

In the present case, the chili peppers were only wiped with a damp cloth and cut on a cutting board. These practices likely failed to eliminate spores and may have contributed to their spread among multiple jars. Additionally, as an oil-based preserve, no acidification with vinegar or adequate salt was applied: these measures could have limited spore germination even under anaerobic conditions. Together, these preparation and storage practices created conditions conducive to spore survival and subsequent toxin production in the affected jar. Furthermore, FB was not immediately suspected considering that at least eight days passed between the initial symptoms and the subsequent severe symptoms, such as dysphagia, dry mouth, ptosis, and diplegia, which could already be defined, delaying the diagnosis. This clearly highlights the importance of medical awareness to limit the seriousness of the illness [7]. Luckily, toxin B has a shorter duration of action and is often associated with milder symptoms compared to toxin A, indeed the patient started to recover, even if he required assisted ventilation, as administering antiserum would have been useless after numerous days since symptom onset. The only option was to wait for the toxin to naturally detach from the acetylcholine receptors, continuing assisted ventilation [7,23]. After the foodborne botulism suspicion, samples were immediately sent to the laboratory for the confirmation; the screening through real-time PCR was fast and sensitive to promptly demonstrate the presence of the *bont/B* gene. Surprisingly, the mouse test was negative for all the samples. This can be explained by several factors: uneven distribution of the bacteria responsible for toxin synthesis among the samples, toxin degradation by an unfavorable pH, proteolytic enzymes unsuitable storage conditions, and the time elapsed before testing. Therefore, the mouse test may have been negative because the toxin was no longer present or active in the tested samples. It should also be noted that even if toxin genes are present, toxin production depends on specific environmental conditions (pH, temperature, anaerobiosis, and nutrients), which can vary slightly from jar to jar and may result in different BoNT production levels. These considerations highlight the limitations of classical detection methods and underscore the value of molecular approaches such as real-time PCR and WGS for outbreak investigations. In fact, the real-time PCR enabled the establishment of the presence of the pathogen’s DNA in the food and the type of toxin that could be produced. However, the successful isolation of bacteria from the human sample and the preserves enabled WGS for further investigations to confirm and trace the contaminated food. The isolates were assigned to two different STs (29 and 53), which were previously described in the literature as being associated with FB outbreaks in Japan and France, respectively [24,25]. Notably in our case, the strain isolated from the intestinal washing of Patient 2 belonged to ST53 as well as the isolates from three preserves (samples 6, 7, and 9). This suggested that the strain that caused severe symptoms in Patient 2 was the same one that contaminated the preserves during the preparation; these strains were also related according to SNP typing. In fact, the isolates from the human intestinal washing and sample 7 isolated from the preserve showed no SNP difference. Owing to SNP typing it was possible to identify the origin of the FB outbreak, despite the absence of residues of the contaminated food that was actually consumed. Moreover, WGS results demonstrated the presence of an additional strain during preparation (ST29) that contaminated one can (sample 4), but as far as we know, it was not ingested by the patients.

Genomic analyses also revealed the presence of the *cfr(C)* AMR gene, encoding for RNA methylase, conferring a multi-resistant phenotype that includes resistance to phenicols, lincosamides, oxazolidinones, pleuromutilins, and streptogramin A compounds [26]. This gene has already been identified in numerous other bacteria, including *Campylobacter* spp. and various *Clostridia* species, such as *C. difficile* and *C. perfringens* [27,28]. Despite antibiotics not being recommended when treating botulism, the presence of AMR genes in *C. botulinum* strains could enhance the probability of transfer of resistance genes between different bacterial species. In particular, *cfr(C)* confers resistance to clindamycin and linezolid, which are antibiotics used to treat serious infections caused by different bacteria, such as vancomycin-resistant enterococci and methicillin-resistant staphylococci; thus, the monitoring of this AMR gene is a significant concern and WGS analysis confirmation to provide broad-spectrum monitoring. However, further studies in the AMR field are needed to better understand the role of genes conferring AMR in *C. botulinum*. These findings demonstrate the high resolution of WGS in tracing FB outbreaks, even when the actual consumed product is unavailable [29].

## 5. Conclusions

This study highlights the importance of collaboration among hospitals, LHAs, and laboratories to ensure the prompt confirmation of suspected foodborne poisoning and to provide appropriate medical care. Timely identification is important for tracing and containing the source of the *C. botulinum* intoxication, thereby preventing further cases.

WGS provided valuable insights into the genetic characteristics of the strains involved, including confirmation of the *bont/B* gene, the *cfr(C)* resistance gene detection, and the identification of different STs, namely ST53 and ST29. These findings underline the importance of investigating and reporting the genomic relationships among *C. botulinum* strains to enhance control measures and improve outbreak management.

## Figures and Tables

**Table 1:** Home-made food products suspected to be associated with the botulism outbreak with the ingredients used for the preparation.

Number of Cans Collected	Ingredients	Sample
2	Chili peppers–not Habanero, oil	1–2
6	Habanero peppers, oil	3–8
1	Habanero peppers, oil, garlic	9

**Table 2:** Main parameters evaluated for the assemblies. N50 denotes the length of the shortest contig that contributes to at least 50% of the total assembly, whereas L50 refers to the minimum number of contigs needed to reach 50% genome coverage. Together, these metrics indicate the assembly quality.

Sample	Contigs ≥ 500 bp	N50	L50	Largest Contig (Mbp)	Total Length (Mbp)
4	24	626,666	2	1.54	3.81
6	25	425,104	4	0.69	3.81
7	21	313,607	2	1.59	3.86
9	23	521,675	2	1.60	3.85
Intestinal washing	24	425,101	3	1.25	3.86

**Table 3:** Strains isolated from the home-made food products and from the intestinal washing of Patient 2, along with the ingredients used for their preparation, the anti-microbial resistance (AMR) genes identified, and the sequence types (STs) determined with MLST.

Sample	Ingredients	ST	AMR Genes
4	Habanero peppers, oil	29	*cfr(C)*
6	Habanero peppers, oil	53	*cfr(C)*
7	Habanero peppers, oil	53	*cfr(C)*
9	Habanero peppers, oil, garlic	53	*cfr(C)*
Intestinal washing	/	53	*cfr(C)*

**Table 4:** SNP distance matrix results for the *C. botulinum* strains, with sample 4 (chili peppers preserve) used as the reference strain.

Samples	4	6	7	9	Intestinal Washing
4	0	14,834	14,841	14,838	14,841
6	14,834	0	125	4	125
7	14,841	125	0	129	0
9	14,838	4	129	0	129
Intestinal washing	14,841	125	0	129	0

## Data Availability

The original contributions presented in the study are included in the article, further inquiries can be directed to the corresponding author.

## Abbreviations

The following abbreviations are used in this manuscript:

BoNT	*Clostridium botulinum* neurotoxin
BoNT/B	*Clostridium botulinum* neurotoxin type B
*BoNT/B*	*Clostridium botulinum* gene encoding for neurotoxin type B
WGS	Whole Genome Sequencing
IZSLER	Istituto Zooprofilattico Sperimentale della Lombardia e dell’Emilia Romagna
LHA	Local Health Authority
CNRB	Italian National Reference Centre for Botulism
CP	Process control
FB	Foodborne botulism
TPGY	Triptone peptone glucose yeast extract
PCR	Polymerase chain reaction
EYA	Egg-yolk agar
AMR	Anti-microbial resistance
ST	Sequence type
PubMLST	Public databases for multi-locus sequence typing
SNP	Single-nucleotide polymorphism
bp	Base pair
